# A Novel tsRNA, m^7^G‐3′ tiRNA Lys^TTT^, Promotes Bladder Cancer Malignancy Via Regulating ANXA2 Phosphorylation

**DOI:** 10.1002/advs.202400115

**Published:** 2024-06-18

**Authors:** Xiaoling Ying, Wenyu Hu, Yapeng Huang, Yifan Lv, Ding Ji, Cong Chen, Baotong Yang, Chengcheng Zhang, Yaomin Liang, Haiqing Zhang, Mingrui Liu, Gang Yuan, Wenqi Wu, Weidong Ji

**Affiliations:** ^1^ Center for Translational Medicine The First Affiliated Hospital Sun Yat‐sen University Guangzhou 510080 China; ^2^ Department of Urology The Second Affiliated Hospital of Guangzhou Medical University Guangzhou 510220 China; ^3^ Guangdong Provincial Key Laboratory of Urology Guangzhou 510230 China; ^4^ Department of Urology The First Affiliated Hospital of Guangzhou Medical University Guangzhou 510230 China; ^5^ Department of Otolaryngology The First Affiliated Hospital Sun Yat‐sen University Guangzhou Guangzhou 510080 China; ^6^ Private Medical Service & Healthcare Center The First Affiliated Hospital Sun Yat‐sen University Guangzhou 510080 China

**Keywords:** ANXA2, Bladder cancer, m^7^G‐3′‐tiRNA Lys^TTT^ (mtiRL), tRNA‐derived fragments, Yes1

## Abstract

Emerging evidence indicates that transfer RNA (tRNA)‐derived small RNAs (tsRNAs), originated from tRNA with high abundance RNA modifications, play an important role in many complex physiological and pathological processes. However, the biological functions and regulatory mechanisms of modified tsRNAs in cancer remain poorly understood. Here, it is screened for and confirmed the presence of a novel m^7^G‐modified tsRNA, m^7^G‐3′‐tiRNA Lys^TTT^ (mtiRL), in a variety of chemical carcinogenesis models by combining small RNA sequencing with an m^7^G small RNA‐modified chip. Moreover, it is found that mtiRL, catalyzed by the tRNA m^7^G‐modifying enzyme mettl1, promotes bladder cancer (BC) malignancy in vitro and in vivo. Mechanistically, mtiRL is found to specifically bind the oncoprotein Annexin A2 (ANXA2) to promote its Tyr24 phosphorylation by enhancing the interactions between ANXA2 and Yes proto‐oncogene 1 (Yes1), leading to ANXA2 activation and increased p‐ANXA2‐Y24 nuclear localization in BC cells. Together, these findings define a critical role for mtiRL and suggest that targeting this novel m^7^G‐modified tsRNA can be an efficient way for to treat BC.

## Graphical abstract

In this study, it specifically shows that the novel m^7^G‐modified tRNA‐derived small RNA (tsRNA) m^7^G‐3′‐tiRNA Lys^TTT^ (mtiRL), catalyzed by the tRNA m^7^G‐modifying enzyme mettl1, specifically binds ANXA2, promoting its Tyr24 phosphorylation, by enhancing ANXA2–Yes1 interactions, leading to ANXA2 activation in bladder cancer (BC) cells and promotes BC malignancy.

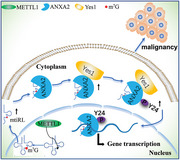

## 1 Introduction

RNA post‐transcriptional modifications, also known as epitranscriptomes, have crucial roles in the biological functions of RNA. More than 170 post‐transcriptional RNA modifications have been identified.^[^ ^1^ ^]^ Transfer RNAs (tRNAs) undergo the most abundant modifications, including methylation, acetylation, pseudouridylation and et al.^[^ ^2^ ^]^ These modifications affect tRNA structure and function, including stability, folding, and decoding properties, as well as translation efficiency and fidelity.^[^ ^3^, ^4^ ^]^ In addition to playing an essential role in the transport of activated amino acids to the ribosome during protein synthesis, tRNAs can be cleaved to form a novel group of small non‐coding RNA, tRNA‐derived small RNAs (tsRNAs), which can be further classified into tRNA halves and tRNA‐derived fragments (tRFs).^[^ ^5^ ^]^

tsRNAs have diverse biological functions, including the stress response, stem cell differentiation, ribosome biosynthesis, and tumorigenesis, and are involved in the mechanisms underlying several biological effects, such as transposon silencing, protein translation, and epigenetic regulation.^[^ ^5^, ^6^ ^]^ tsRNAs are present in all organisms, have highly conserved structures, and exhibit marked tissue‐specific expression.^[^ ^7^ ^]^ Among them, the expression of tRNA halves is closely related to the environmental stress response. Studies have demonstrated that angiogenin (ANG) cleaves mature tRNAs at the anticodon loop under various stress conditions, producing tRNA halves with a length of 30–45 nucleotides, also known as tRNA‐derived stress‐induced small RNA (tRNA‐derived stress‐induced small RNA, tiRNA). They can be further divided into 5′‐tiRNA and 3′‐tiRNA according to their corresponding mature tRNA sequence.^[^ ^8^ ^]^ Compared to expression in normal cells, many tsRNAs are expressed differently in tumor cells and are key players in the development and progression of cancer. For example, hypoxia‐induced tRF‐Asp, tRF‐Glu and tRF‐Gly inhibit breast cancer cell metastasis by competitively binding to YBX‐1.^[^ ^9^ ^]^ Moreover, tRF‐21, regulated by inflammatory factors combined with hnRNP L, suppresses pancreatic cholangiocarcinoma progression.^[^ ^10^ ^]^ Meanwhile 5′‐tiRNA‐His‐GTG promotes colorectal cancer progression by downregulating *LATS2* mRNA translation.^[^ ^11^ ^]^ Recent studies have also indicated that modified tsRNAs are involved in the occurrence of disease. Endogenous m^5^C‐ and m^2^G‐modified tsRNAs in the sperm of male mice fed a high‐fat diet led to metabolic disorders in F1 offspring, whereas unmodified synthetic tsRNAs have no effect.^[^ ^12^ ^]^ Furthermore, in bladder urothelial carcinoma, high expression of the TRMT6/TRMT61A methyltransferase complex upregulates the m^1^A level of tRF‐3b and attenuates tRF‐3b‐mediated gene silencing, which is crucial for maintaining a steady‐state unfolded protein response.^[^ ^13^ ^]^ Moreover, tRFs with pseudouridine (Ψ) modifications inhibit abnormal protein synthesis and promote the progression of myelodysplastic syndrome.^[^ ^14^ ^]^ However, the role of modified tsRNAs in tumorigenesis and development and their regulatory mechanisms are poorly understood.

Our previous study showed that the level of METTL1 is dramatically increased in bladder cancer (BC) tissues, and high expression of this marker was found to be associated with BC invasion.^[^ ^15^ ^]^ In humans, the METTL1/WDR4 complex catalyzes the formation of 7‐methylguanosine (m^7^G) at the 46^th^ position of the tRNA variable loop. Recent studies have also shown that the METTL1/WDR4 complex can enhance the translation of cell cycle genes (such as *CCNA2*, *CCND2*, *CDK6*, and *CDK8*) and oncogenes (such as *EGFR*, *EFEMP1*, and *KDM1A*), leading to the development of gliomas, pancreatic cancer, liver cancer, lung cancer, BC, and other tumors.^[^ ^15^, ^16^, ^17^, ^18^, ^19^ ^]^ Thus, METTL1/WDR4‐mediated tRNA m^7^G modification plays a key role in tumor development. However, the role of m^7^G‐modified tsRNAs in tumorigenesis remains unclear.

In this study, we screened for and identified a novel m^7^G‐modified tsRNA, m^7^G‐3′‐tiRNA Lys^TTT^ (mtiRL), which is involved in chemical carcinogenesis and is catalyzed by METTL1. In vitro and in vivo experiments demonstrated that high mtiRL expression facilitates BC malignancy and has an oncogenic role. Mechanistically, we found that mtiRL specifically binds to the important carcinogenic protein Annexin A2 (ANXA2) and enhances the binding between Yes proto‐oncogene 1 (Yes1) and ANXA2, thereby promoting the capacity of Yes1 to phosphorylate ANXA2 at the Tyr24 site. Kaplan–Meier survival analysis further demonstrated that high expression of p‐ANXA2‐Y24 in BC is associated with poor prognosis. This study not only reveals an RNA epigenetic mechanism of bladder cancer development, but also provides strategies to explore novel early biomarkers of tumorigenesis.

## 2 Results

### 2.1 Identification of a Novel m^7^G‐Modified tsRNA, m^7^G‐3′‐tiRNA Lys^TTT^, in Transformed Urothelial Cells and BC Cells

**Figure 1:**
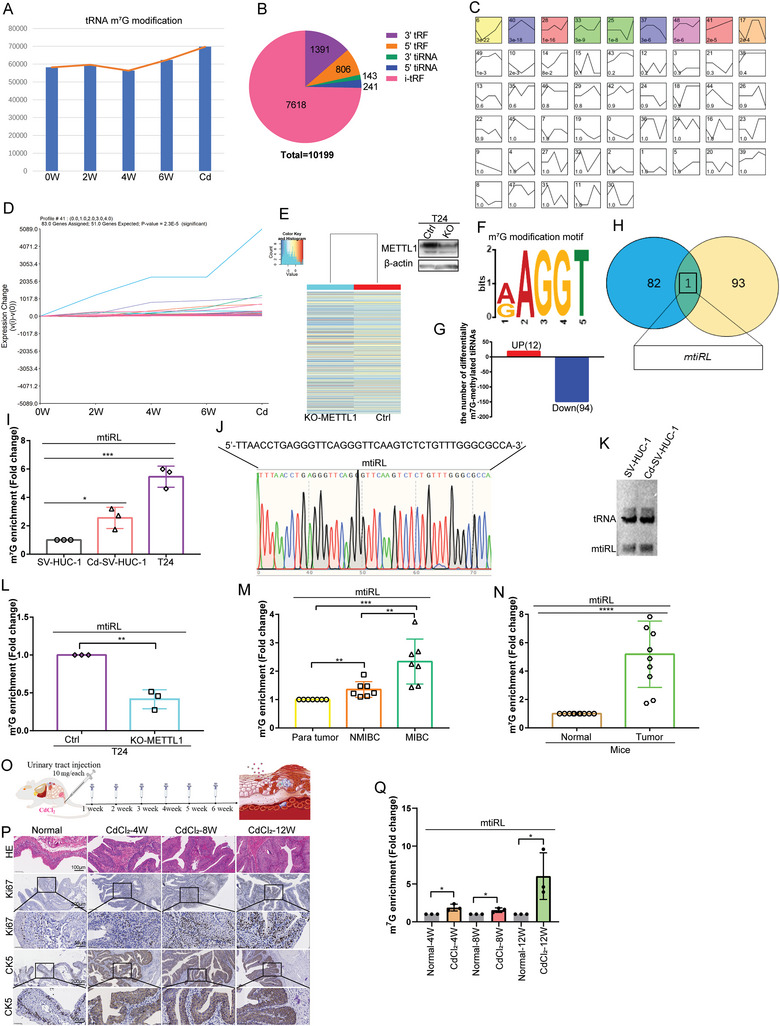
High expression of mtiRL involved in bladder cancer malignancy. A) tRNA modification was analyzed using LC/MS in multistage CdCl_2_ malignant transformed cells. B) Small RNA sequencing in multistage CdCl_2_ malignant transformed cells. C) Trend analysis of tRFs & tiRNAs. D) Model 41 in which the expression of tRFs & tiRNAs was increased upon the prolonging CdCl_2_ treatment time. E) Analyzed expression of m^7^G‐tRF&tiRNA using Arraystar Human m^7^G small RNA modification microarray in METTL1‐knockout T24 cells and control cells. F) m^7^G motif tested in Arraystar Human m^7^G small RNA modification microarray. G) 12 tRFs&tiRNAs containing m^7^G motif were significantly up‐regulated, while 94 containing m^7^G motif significantly down‐regulated after METTL1‐knockout. H) A Venn analysis on tRFs&tiRNAs in trend analysis model 41 and downregulated tRFs&tiRNAs. I) The abundance of m^7^G −3′tiRNA Lys^TTT^‐10 (mtiRL) were detected in SV‐HUC‐1, Cd‐SV‐HUC‐1 and T24 cells by m^7^G immunoprecipitation‐ Stem‐loop RT PCR method. J) The Sanger sequencing of the PCR products mtiRL. K) Northern blot further confirmed the expression of mtiRL in SV‐HUC‐1 and Cd‐SV‐HUC‐1 cells. L) The results indicated that mtiRL decreased significantly in knockout METTL1 T24 cells. M) The expression of mtiRL was tested in para‐tumor (n = 7), NMIBC(n = 7) and MIBC samples(n = 7). N) The level of mtiRL was measured in 10 bladder tumor tissues from Benzopyrene‐induced multiple organ mice carcinogenesis models. O) Multi‐stage models of carcinogenesis using CdCl_2_ were constructed. P) Bladder cancer subtypes were determined by HE staining and the IHC expressions of Ki67and urothelial lineage markers CK5. Q) The expression of mtiRL in multi‐stage bladder tissues was tested. ^*^ *p* < 0.05, ^**^ *p* < 0.01, ^***^ *p* < 0.001, ^****^ *p* < 0.0001.

To determine whether tRNA modification plays a role in the malignant transformation of bladder epithelial cells, we measured the levels of 58 different tRNA modifications during carcinogen CdCl_2_‐induced urothelial cell transformation using liquid chromatography–mass spectrometry (LC‐MS) (Figure S1A, Supporting Information). The results showed that the level of tRNA m^7^G modification increased with the extension of CdCl_2_ treatment (**Figure** **1**A; Figure S1B, Supporting Information), suggesting that it is involved in the carcinogenesis of bladder epithelial cells. Under stress conditions, including chemical carcinogen exposure, ANG divides mature tRNAs into two halves (3′‐tiRNA and 5′‐tiRNA) at the anticodon loop.^[^ ^20^ ^]^ tiRNAs are highly expressed in multiple tumor tissues and play key roles in tumor carcinogenesis.^[^ ^11^, ^21^, ^22^, ^23^, ^24^ ^]^ Thus, we hypothesized that m^7^G‐3′‐tiRNA, derived from m^7^G‐tRNA cut by ANG, might have a critical role in the transformation of urothelial cells. To identify malignant transformation‐related m^7^G‐tiRNAs, we first detected tsRNA expression in CdCl_2_‐transformed malignant cells (Cd‐SV‐HUC‐1 cells) and multistage CdCl_2_‐treated urothelial cells (SV‐HUC‐1 cells) at 0, 2, 4, and 6 weeks via small RNA sequencing. Here, 10 199 tRNA fragments were found in the five aforementioned cell lines (Figure 1B); of them, 143 3′‐tiRNAs and 241 5′‐tiRNA were identified (Figure 1B). Differentially expressed tRFs and tiRNAs (fold‐change (FC) ≥ 2 or < 0.5 and *p*‐value < 0.05) were also observed in the CdCl_2_‐treated cell lines (Figure S2A–D, Supporting Information). We then conducted a trend analysis, which indicated significance in 9 (models 6, 40, 28, 33, 25, 37, 48, 41, and 17) of 50 models (*p* < 0.05; Figure 1C). In model 41, 83 total tRFs and tiRNAs were detected, of which, the expression was increased upon prolonged CdCl_2_ treatment (Figure 1D). We further analyzed the expression of m^7^G‐tRF and tiRNA using an Arraystar Human m^7^G small‐RNA modification microarray using METTL1‐knockout T24 cells and control cells. Here, 3540 m^7^G‐tRFs and tiRNAs were detected. Of them, expression levels of 293 were significantly upregulated (FC > 2) and those of 526 m^7^G‐tRFs and tiRNAs were downregulated (FC < 0.5) (Figure 1E). Only 12 types were found to contain an m^7^G motif among significantly upregulated tRFs and tiRNAs, whereas 94 significantly downregulated tRFs and tiRNAs contained the m^7^G motif (Figure 1F,G). Furthermore, we conducted a Venn analysis of tRFs and tiRNAs from the trend analysis of model 41 and downregulated tRFs and tiRNAs containing an m^7^G motif. Finally, we obtained m^7^G‐3′‐tiRNA Lys^TTT^ (mtiRL) (Figure 1H).

### 2.2 High Expression of mtiRL is Involved in BC Malignancy

As tiRNA genes are short and overlap with the parental tRNA, conventional PCR cannot distinguish signals among tRNA halves (tiRNAs), mature tRNA, and pre‐tRNA. Therefore, we developed a stem‐loop RT‐PCR method that can specifically quantify the expression of 3′‐tiRNA (Figure S3A, Supporting Information). We first tested the expression of 3′‐tiRNA Lys^TTT^ (tiRL) in SV‐HUC‐1 and Cd‐SV‐HUC‐1 cells using this method. Based on the results, tiRL, but not tRNA Lys^TTT^, signals could be detected (Figure S3B, Supporting Information). We further examined the abundance of mtiRL in SV‐HUC‐1, Cd‐SV‐HUC‐1, and T24 cells via m^7^G immunoprecipitation (IP)‐stem‐loop RT‐PCR. The results demonstrated that mtiRL was significantly enriched in Cd‐SV‐HUC‐1 and T24 cells compared to that in control SV‐HUC‐1 cells (Figure 1I). Moreover, we conducted Sanger sequencing of the obtained PCR products, and the sequences matched perfectly (Figure 1J). Northern blotting confirmed mtiRL expression (Figure 1K) in METTL1‐deficient T24 cells and empty vector control cells, and the results indicated that mtiRL expression was significantly decreased in METTL1‐deficient T24 cells (Figure 1L). Furthermore, we detected an abundance of mtiRL in seven groups of normal bladder urothelial, non‐muscle invasive bladder cancer (NMIBC), and muscle invasive bladder cancer (MIBC) tissues. The results showed that mtiRL expression was higher in NMIBC and MIBC samples than in normal samples (Figure 1M). Further, MIBC tissues exhibited the highest expression levels (Figure 1M). In addition, the sequence of 3′tiRNA‐Lys^TTT^ is highly conserved between human, mouse, and rat (Table S3, Supporting Information). We generated mouse models of benzopyrene‐induced multiple‐organ carcinogenesis (Figure S4, Supporting Information) and collected 10 bladder tumor tissues. The samples were then analyzed using m^7^G IP–stem‐loop RT‐PCR, and the results implied that mtiRL was expressed at high levels in cancer cells (Figure 1N). To further explore the association between mtiRL and BC, we constructed multi‐stage models of carcinogenesis using CdCl_2_. The initial state of the cells was normal, with no presence of cell atypia and a scattered arrangement. There was no evidence of cell proliferation. However, after 4 weeks of bladder perfusion, the group treated with CdCl_2_ showed abnormal cell proliferation accompanied by infiltration of inflammatory cells. By the eighth week, this group exhibited cell dysplasia, characterized by the presence of mitotic figures. After 12 weeks, there was an observed increase in both the number of cell layers and cell density (Figure 1O). Bladder cancer subtypes were determined by the IHC expressions of Ki67 and CK5 (Figure 1P). The IHC data showed that our multi‐stage modeling was successful. Then the expression of mtiRL in multi‐stage bladder tissues was tested. The results indicated that the level of mtiRL in the modeling group were gradually enhanced with the degree of malignancy (Figure 1Q). Taken together, the data demonstrate that the overexpression of mtiRL is involved in BC malignancy.

### 2.3 mtiRL Promotes the Proliferation and Migration of BC Cells

**Figure 2:**
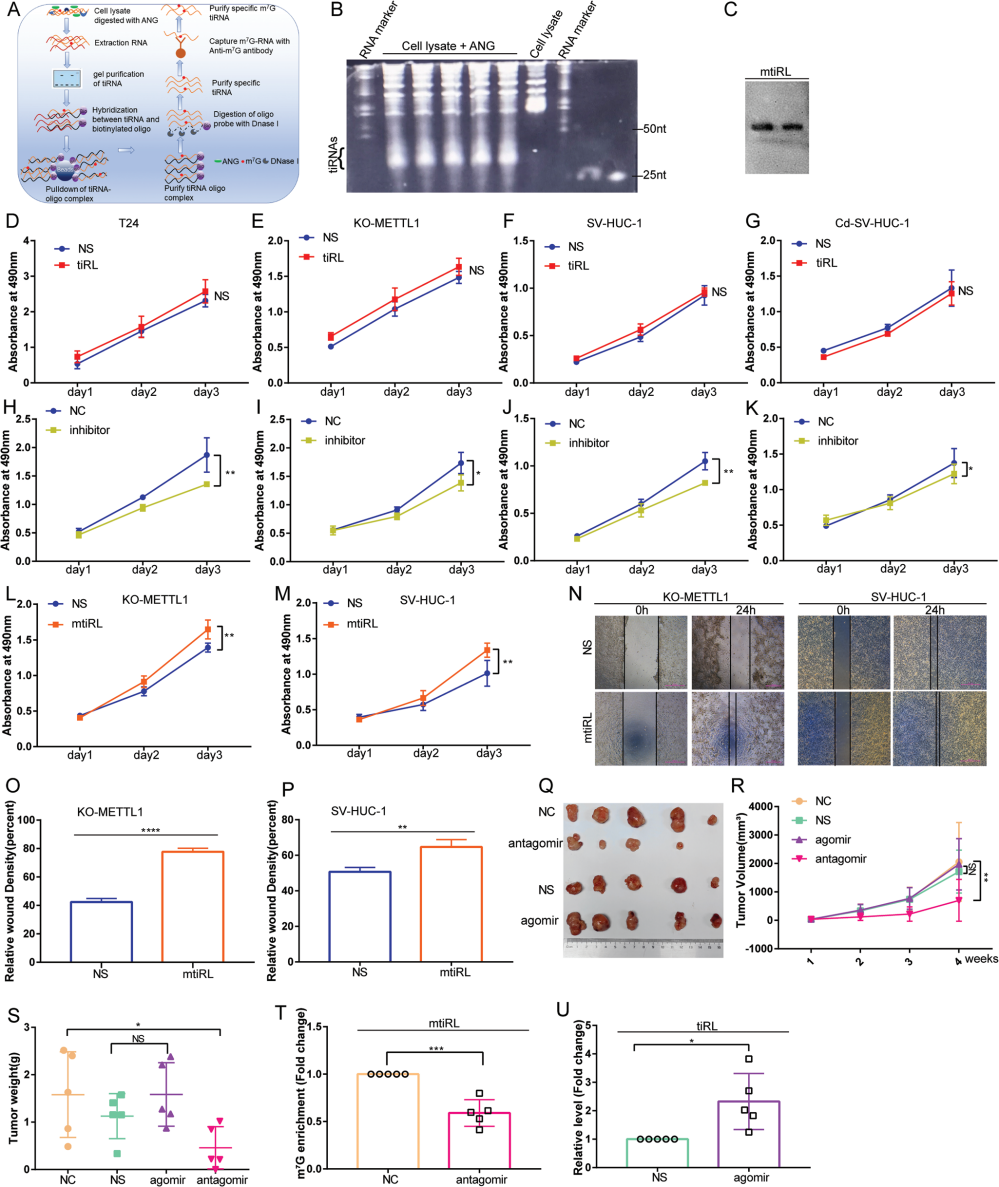
mtiRL promoted proliferation and migration of bladder cancer. A) Schematic illustration of extracting endogenous mtiRL. B) tiRNAs were separated on 12% TBE‐urea polyacrylamide gels. C) Captured endogenous mtiRL was verified by Northern blot. D–G) Cell proliferation assay showed that tiRL (without m^7^G modification) overexpression does not affect cell proliferation in T24 cells, METTL1‐deficient T24 cells, SV‐HUC‐1 and Cd‐SV‐HUC‐1 cells. H–K) The proliferative capacity of cells was significantly down‐regulated by small interfering RNA in T24 cells, METTL1‐deficient T24 cells, SV‐HUC‐1 and Cd‐SV‐HUC‐1 cells. L,M) Overexpression of endogenous mtiRL significantly upregulated the ability of proliferation in METTL1deficient T24 and SV‐HUC‐1 cells. N–P) Overexpression of endogenous mtiRL significantly upregulated the ability of migration in METTL1deficient T24 and SV‐HUC‐1 cells. Q–S) Both tumor volume (R) and tumor weight (S) were markedly decreased with antagomir treatment compared to NC antagomir group, whereas 3′tiRNA Lys^TTT^ overexpression did not influence tumor growth. (Each group n = 5). T) Antagomir treatment significantly reduced m^7^G‐3′tiRNA Lys^TTT^ expression. U) agomir treatment considerably increased 3′tiRNA Lys^TTT^ expression. ^*^ *p* < 0.05, ^**^ *p* < 0.01, ^***^ *p* < 0.001, ^****^ *p* < 0.0001.

To investigate the role of mtiRL in BC, we obtained endogenous mtiRL using a complementary DNA oligonucleotide probe combined with m^7^G IP (**Figure** **2**A,B). Captured mtiRL was verified by performing northern blotting (Figure 2C). Additionally, tiRL, without m^7^G modification, was synthesized and transfected into T24, METTL1‐deficient T24, SV‐HUC‐1, and Cd‐SV‐HUC‐1 cells. The cell proliferation assay showed that tiRL overexpression did not affect the proliferation of these cell lines (Figure 2D–G). However, when mtiRL expression was inhibited by small interfering RNA (siRNA), the proliferative capacity of the cells was significantly decreased (Figure 2H–K). Notably, we found that the overexpression of endogenous mtiRL significantly increased the proliferation and migration abilities of METTL1‐deficient T24 and SV‐HUC‐1 cells (Figure 2L–P). These results suggest that mtiRL, but not tiRL, promotes BC malignancy. To confirm these in vivo results, 2 × 10^6^ T24 cells were subcutaneously injected into nude mice; 1 week later, the tumors were treated with the NC antagomir, tiRL antagomir, NS agomir, or tiRL agomir via multi‐site intratumoral injection every 3 days, five times. Tumor volumes were measured weekly, and the tumors were extracted after 4 weeks. With antagomir treatment, both tumor volumes and weights were markedly decreased compared to those in the NC antagomir group, whereas tiRL overexpression did not influence tumor growth (Figure 2Q–S). In addition, we measured the levels of mtiRL in the antagomir‐treated group and tiRL in the agomir‐treated group. The results showed that antagomir treatment significantly reduced mtiRL expression, whereas agomir treatment considerably increased tiRL expression (Figure 2T,U). Overall, our in vitro and in vivo assays indicated that mtiRL plays an oncogenic role in BC.

### 2.4 mtiRL Directly Binds to ANXA2

**Figure 3:**
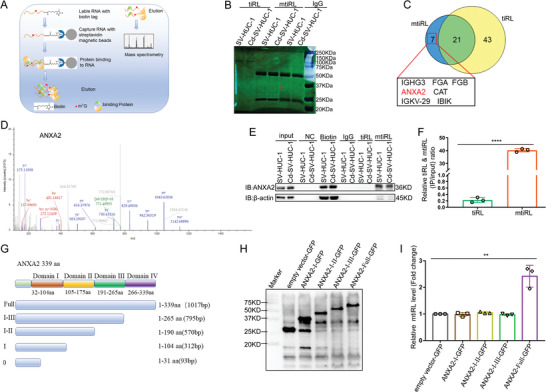
mtiRL directly binds to ANXA2. A) Schematic illustration of endogenous mtiRL pull down. B) SDS‐PAGE of pull‐down proteins. C) Venny analysis of mtiRL and tiRL pull‐down proteins. D) Protein profile of ANXA2. E) mtiRL could specifically pull down ANXA2 protein both in SV‐HUC‐1 and Cd‐SV‐HUC‐1 cells. F) RIP assays indicated that ANXA2 interacts with mtiRL. G) Domain diagram of ANXA2. H) WB detected protein expression of vectors carrying GFP‐tagged full‐length and truncated ANXA2 Flag‐tagged ANXA2. I) RNA immunoprecipitation (RIP) assays demonstrated that mtiRL interacts with the domain IV of ANXA2, but not others. All qRT‐PCR data are presented as means ±SEM. n = 3. ^**^ *p* < 0.0001, ^****^ *p* < 0.0001.

To determine the oncogenic mechanism of mtiRL, we extracted endogenous mtiRL and captured proteins binding to it through biotin‐labeled RNA pull‐down assays, followed by tandem mass spectrometry (MS) to analyze the binding proteins (**Figure** **3**A); 28 proteins were identified in the labeled mtiRL‐probe group, and 64 proteins were detected in the labeled tiRL‐probe group (Figure 3B,C). We selected seven proteins that bound to mtiRL but not tiRL (Figure 3C,D). The functions and structures of these proteins were further analyzed; among them, we found that ANXA2 contains RNA‐binding sites^[^ ^25^ ^]^ and plays an important role in various cancers, including BC.^[^ ^25^, ^26^, ^27^, ^28^, ^29^, ^30^ ^]^ To further validate the binding of mtiRL to ANXA2, RNA pull‐down and RNA IP assays coupled with m^7^G IP were performed. The results revealed that mtiRL could specifically pull down the ANXA2 protein in both SV‐HUC‐1 and Cd‐SV‐HUC‐1 cells (Figure 3E). An RNA immunoprecipitation (RIP) assay using anti‐ANXA2 indicated that ANXA2 interacted with mtiRL (Figure 3F). To further interrogate the exact domain between ANXA2 and mtiRL, we constructed vectors carrying GFP‐tagged full‐length (domain I–IV (1‐339aa)) and truncated ANXA2 (domain I (1‐104aa), domain I–II (1‐190aa), domain I–III (1‐265aa)) (Figure 3G; Figure S5, Supporting Information). Each vector was individually transfected into 293T cells, followed by IP with anti‐GFP. Transfected proteins were verified by western blotting (Figure 3H). Moreover, the results of RNA immunoprecipitation (RIP) assays demonstrated that mtiRL interacts with the domain IV, but not others (Figure 3I). These results suggest that ANXA2 is a direct target for mtiRL mediated BC tumorigenesis.

### 2.5 mtiRL Increases the Phosphorylation of ANXA2 at Tyr24

**Figure 4:**
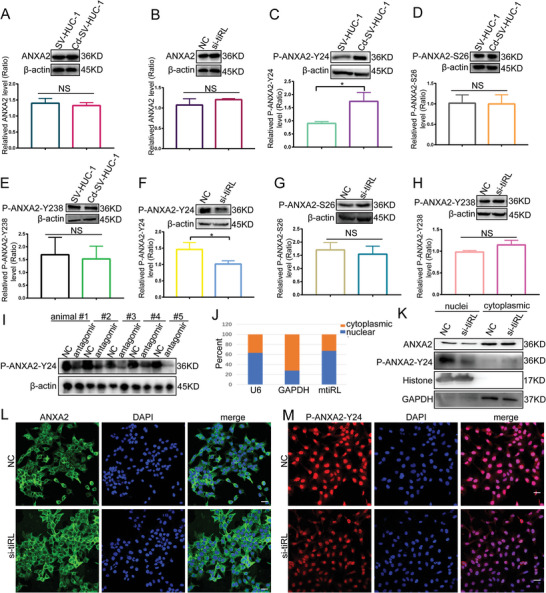
mtiRL increased the expression of p‐ANXA2 at Tyr24 site. A) WB results revealed that no significant difference in expression levels of ANXA2 between SV‐HUC‐1 and Cd‐SV‐HUC‐1 cells. B) ANXA2 level remained unchanged when mtiRL expression was downregulated by small interfering RNA in Cd‐SV‐HUC‐1 cells. C) WB results showed that the level of Tyr24 phosphorylation of ANXA2 (p‐ANXA2‐Y24) is up‐regulated in Cd‐SV‐HUC‐1 cells. D,E) The levels of Ser26 phosphorylation of ANXA2 (p‐ANXA2‐S26) and Tyr238 phosphorylation of ANXA2(p‐ANXA2‐Y238) were unchanged. F) The level of p‐ANXA2‐Y24 decreased with mtiRL downregulation. G,H) The expressions of p‐ANXA2‐S26 and p‐ANXA2‐Y238 remained unaltered after mtiRL downregulation. I) WB results indicated that mtiRL interfering RNA significantly downregulated p‐ANXA2‐Y24 expression in mice tumor tissues treated with antagomir. J) The results of nucleocytoplasmic separation experiment showed that mtiRL mostly distributed in nuclei. K) Nucleocytoplasmic separation assays demonstrated that ANXA2 mainly localized in cytoplasmic, while p‐ANXA2‐Y24 largely distributed in nucleus. The expression of p‐ANXA2‐Y24 reduced in Cd‐SV‐HUC‐1 cells treated with mtiRL interfering RNA. L,M) The results of immunofluorescence assays were consistent with that of nucleocytoplasmic separation assays. (Scale bar, 100 µm), ^*^ *p* < 0.05.

Next, we explored the effects of mtiRL binding to ANXA2. The expression of ANXA2 in SV‐HUC‐1 and Cd‐SV‐HUC‐1 cells was measured by performing western blotting, which revealed no significant difference in ANXA2 expression levels between SV‐HUC‐1 and Cd‐SV‐HUC‐1 cells (**Figure** **4**A). Furthermore, we downregulated mtiRL expression using siRNA in Cd‐SV‐HUC‐1 cells and found that ANXA2 levels remained unchanged (Figure 4B). Recent studies have shown that ANXA2 is activated by phosphorylation and is involved in cancer.^[^ ^28^ ^]^ To investigate the effect of mtiRL on ANXA2 phosphorylation, the protein levels of ANXA2 phosphorylated at Tyr24 and Ser26, the two main phosphorylation sites of ANXA2^[^ ^31^ ^]^ were detected through western blotting. Tyr24 phosphorylation of ANXA2 (p‐ANXA2‐Y24) was increased in Cd‐SV‐HUC‐1 cells (Figure 4C). However, the level of Ser26 phosphorylation of ANXA2 (p‐ANXA2‐S26) remained unchanged (Figure 4D). We also detected the Tyr238 phosphorylation of ANXA2 (p‐ANXA2‐Y238) and found that the level did not change (Figure 4E). Notably, the level of p‐ANXA2‐Y24 decreased concomitantly with the downregulation of mtiRL expression, whereas the levels of p‐ANXA2‐S26 and p‐ANXA2‐Y238 remained unaltered (Figure 4F–H). In addition, we validated the expression of p‐ANXA2‐Y24 in mouse tumor tissues treated with the antagomir. The results indicated that mtiRL‐interfering RNA could significantly downregulate p‐ANXA2‐Y24 expression (Figure 4I). Moreover, p‐ANXA2‐Y24 is primarily localized in the nucleus.^[^ ^28^ ^]^ We first examined the localization of mtiRL in Cd‐SV‐HUC‐1 cells and found that it was mostly distributed in the nucleus (Figure 4J). Nucleocytoplasmic separation assays were performed using Cd‐SV‐HUC‐1 cells transfected with either negative control RNA or mtiRL‐interfering RNA, and the results demonstrated that ANXA2 was mainly localized in the cytoplasm, whereas p‐ANXA2‐Y24 was largely distributed in the nucleus (Figure 4K). In addition, p‐ANXA2‐Y24 expression was reduced in Cd‐SV‐HUC‐1 cells treated with mtiRL‐interfering RNA, whereas ANXA2 levels remained unchanged (Figure 4K). Immunofluorescence assays were also performed to confirm these results, which were consistent with those of the nucleocytoplasmic separation assays (Figure 4L,M). Together, these results suggest that mtiRL binding to ANXA2 increases the expression of p‐ANXA2‐Y24.

### 2.6 mtiRL Enhances the Binding Between Yes1 and ANXA2

**Figure 5:**
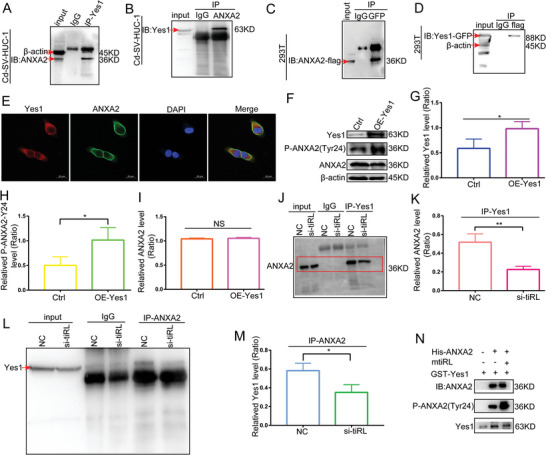
mtiRL enhances the binding between Yes1 and ANXA2. A,B) Endogenous Co‐immunoprecipitation (co‐IP) assays revealed that Yes1 bound to ANXA2. C,D) Exogenous co‐IP assays confirmed that GFP‐Yes1 interacted with Flag‐ ANXA2. E) Colocalization of Yes1 and ANXA2 was confirmed by immunofluorescence staining in Cd‐SV‐HUC‐1 cells. F–I) WB results showed that overexpression of Yes1 upregulated the level of p‐ANXA2‐Y24, whereas it did not affect the expression of total ANXA2 protein. J–M) Co‐IP experiments in Cd‐SV‐HUC‐1 cells revealed that the interaction between endogenous Yes1 and ANXA2 was significantly diminished by knockdown of mtiRL. N) In vitro kinase assay indicated that active GST‐Yes1 could phosphorylate ANXA2 at Tyr24 site and mtiRL enhance the phosphorylation of Yes1. ^*^ *p* < 0.05, ^**^ *p* < 0.01.

The non‐receptor tyrosine kinase Yes1 phosphorylates ANXA2 at Tyr24 in gastric cancer (GC) cells.^[^ ^28^ ^]^ To determine whether Yes1 and ANXA2 interact in Cd‐SV‐HUC‐1 cells, co‐immunoprecipitation (co‐IP) assays were performed. The endogenous co‐IP results revealed that Yes1 bound ANXA2 (**Figure** **5**A,B), whereas exogenous co‐IP assays further confirmed that GFP‐Yes1 interacted with Flag‐ ANXA2 (Figure 5C,D).

Immunofluorescence analysis demonstrated co‐localization of Yes1 to ANXA2 (Figure 5E). The level of p‐ANXA2‐Y24 was also examined in Yes1‐overexpressing Cd‐SV‐HUC‐1 cells using western blotting. The results indicated that the overexpression of Yes1 resulted in the upregulation of p‐ANXA2‐Y24 levels but did not affect the expression of total ANXA2 protein (Figure 5F–I). To examine whether mtiRL enhances the binding between Yes1 and ANXA2, we conducted co‐IP experiments in Cd‐SV‐HUC‐1 cells transfected with siRNA against mtiRL. The results revealed that the interaction between endogenous Yes1 and ANXA2 was significantly diminished by the knockdown of mtiRL (Figure 5J–M). We further tested whether mtiRL increases the capacity of Yes1 to phosphorylate ANXA2 at Tyr24. Results of an in vitro kinase assay indicated that active GST‐Yes1 could phosphorylate ANXA2 at the Tyr24 site and that mtiRL enhanced the phosphorylation ability of Yes1 to ANXA2 (Figure 5N). These results demonstrate that mtiRL upregulates the capacity of Yes1 to phosphorylate ANXA2 at Tyr24 by enhancing its binding to ANXA2.

### 2.7 ANXA2 Restores the Proliferation and Migration of mtiRL‐Knockdown Cells

**Figure 6:**
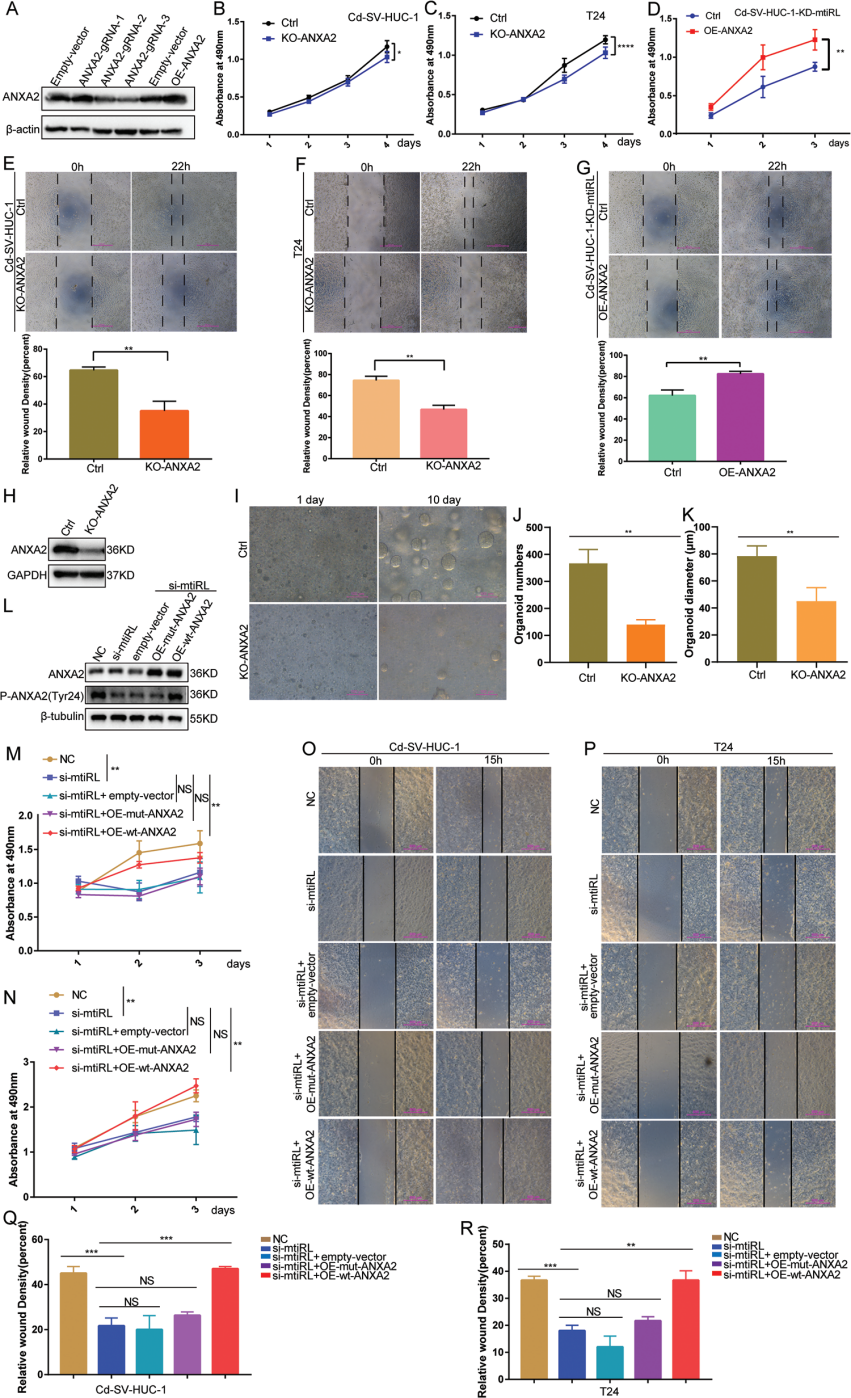
ANXA2 restored proliferation and migration of mtiRL knock down cells. A). Knockout and overexpression of ANXA2 in Cd‐SV‐HUC‐1 cells were detected by WB. B,C) Cellular proliferation demonstrated that ANXA2 depletion suppressed cell proliferation. D) Over‐expression ANXA2 restored the proliferation of mtiRL‐KD cells. E,F) Scratch assays demonstrated that ANXA2 depletion suppressed cell migration ability. G) Over‐expression ANXA2 restored the migration of mtiRL‐KD cells. H–K) ANXA2 knockdown by lentivirus reduces the numbers and size of organoids from models of carcinogenesis using CdCl_2_. L) The wildtype (WT‐ANXA2) or MT‐ANXA2‐Y24F vectors were transfected into mtiRL‐KD cells M) Over‐expression WT‐ANXA2 but not MT‐ANXA2‐Y24F reverted the proliferation of mtiRL‐KD Cd‐SV‐HUC‐1 cells. N) Over‐expression WT‐ANXA2 but not MT‐ANXA2‐Y24F reverted the proliferation of mtiRL‐KD T24 cells. N–Q) Over‐expression WT‐ANXA2 but not MT‐ANXA2‐Y24F reverted migration phenotypes in both mtiRL‐KD Cd‐SV‐HUC‐1 cells (N,P) and mtiRL‐KD T24 cells (O,Q). ^*^ *p* < 0.05, ^**^ *p* < 0.01, ^***^ *p* < 0.001, ^****^ *p* < 0.0001.

In order to investigate the potential role of mtiRL in promoting the proliferation and migration of BC cells through the regulation of ANXA2, we conducted a series of experiments. Initially, we disrupted ANXA2 in Cd‐SV‐HUC‐1 and T24 cells, and subsequently, we overexpressed ANXA2 in mtiRL‐knockdown (KD) Cd‐SV‐HUC‐1 cells (**Figure** **6**A). Through cellular proliferation and scratch assays, we observed that the depletion of ANXA2 resulted in a suppression of cell proliferation and migration. Conversely, the overexpression of ANXA2 restored the proliferation and migration capabilities of mtiRL‐KD cells (Figure 6B–G). In parallel, Bladder cancer organoids were generated from CdCl_2_‐induced bladder cancer models. We knockdown the expression of ANXA2 in organoids and found that both organoid number and size decreased in ANXA2‐KD organoids compared to the control group (Figure 6H–K). To further examine whether the p‐ANXA2‐Y24 recovered their phenotypes of mtiRL‐KD cells, we established mutant‐type ANXA2 (MT‐ANXA2‐Y24F) plasmids. And then the wildtype (WT‐ANXA2) or MT‐ANXA2‐Y24F vectors were transfected into mtiRL‐KD cells. The results of Western blot showed that mutant‐type ANXA2 (MT‐ANXA2‐Y24F) and the wildtype ANXA2 (WT‐ANXA2) were successful overexpression in mtiRL‐KD cells. Furthermore, overexpression of WT‐ANXA2 restored the level of p‐ANXA2‐Y24, while the ANXA2 mutant did not (Figure 6L). Cell proliferation and migration assays were conducted, and the results demonstrated that WT‐ANXA2 but not MT‐ANXA2‐Y24F reverted the proliferation (Figure 6M,N) and migration (Figure 6O–R) phenotypes in both mtiRL‐KD Cd‐SV‐HUC‐1 cells and mtiRL‐KD T24 cells, suggesting that the Tyr24 phosphorylation of ANXA2 can recovered the proliferation and migration abilities of mtiRL‐KD cells. Overall, these findings provide evidence that mtiRL plays a role in enhancing the proliferation and migration of BC cells by modulating ANXA2.

### 2.8 A High p‐ANXA2‐Y24 Expression Level is Associated with a Poor Prognosis

**Figure 7:**
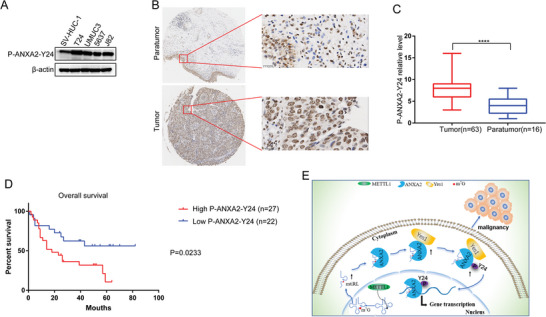
High expression level of p‐ANXA2‐Y24 is associated with a poor prognosis. A). The levels of p‐ANXA2‐Y24 in various bladder cancer cell lines and SV‐HUC‐1 cells were detected by WB. B,C) The level of p‐ANXA2‐Y24 was up‐regulated in tumor tissues compared with para‐cancer normal BC tissues. D) Kaplan–Meier survival analysis demonstrated that BC patients with high p‐ANXA2‐Y24 expression exhibited a worse prognosis than patients with low level of p‐ANXA2‐Y24. E) Summary of mtiRL promotes bladder cancer malignancy via regulating ANXA2 phosphorylation. ^****^ *p* < 0.0001.

We first tested p‐ANXA2‐Y24 expression in various BC cell lines and found that its level was higher than that in SV‐HUC‐1 cells (**Figure** **7**A). We further evaluated p‐ANXA2‐Y24 expression using a human tissue microarray from 63 patients with BC. The results showed that the level of p‐ANXA2‐Y24 was upregulated in tumor tissues compared to that in paracancerous normal BC tissues (Figure 7B,C). Kaplan–Meier survival analysis demonstrated that patients with BC with high p‐ANXA2‐Y24 expression exhibited poorer overall survival than those with low levels of p‐ANXA2‐Y24 (Figure 7D). These results showed that high p‐ANXA2‐Y24 expression in BC is correlated with poor prognosis.

## 3 Discussion

Currently, the functional role of m^7^G‐modified tsRNAs in tumors is unknown. In the present study, we identified mtiRL as a novel m^7^G‐modified tsRNA in transformed urothelial and BC cells for the first time and found that mtiRL was highly expressed in BC and involved in BC malignancy. Our in vitro and in vivo assays further indicated that mtiRL promotes BC growth. To probe the underlying mechanism, we performed a series of experiments and found that mtiRL specifically interacts with ANXA2 and increases the expression of p‐ANXA2‐Y24, but not total ANXA2. We further showed that mtiRL can enhance the binding between ANXA2 and Yes1, which phosphorylates ANXA2 at Tyr24. Collectively, these results describe the functional roles and regulatory mechanism of mtRL, which has an oncogenic role in BC.

ANXA2, also known as annexin A2, is a multifunctional protein that participates in various biological functions, such as endocytosis, exocytosis, actin remodeling, signal transduction, protein assembly, transcription, and mRNA transportation, as well as DNA replication and repair.^[^ ^32^ ^]^ Further, research has shown that it plays an important role in the occurrence and development of head and neck cancer, lung cancer, liver cancer, BC, and other solid tumors by regulating the cell cycle, cell proliferation, fibrinolysis, cytoskeleton reconstruction, and other pathways.^[^ ^32^, ^33^ ^]^ ANXA2 not only forms a heterodimer with the S100A10 protein to activate the carcinogenesis‐associated plasminogen receptor pathway, but also activates signaling pathways, such as STAT3 and Wnt/β‐catenin, through calcium‐binding rings, RNA‐binding sites, and phosphorylation sites in different domains to promote tumor development.^[^ ^28^, ^31^, ^34^, ^35^ ^]^ To test whether mtiRL affects the binding between ANXA2 and S100A10, we performed co‐IP experiments using Cd‐SV‐HUC‐1 cells transfected with siRNA against mtiRL and found that the interaction between ANXA2 and S100A10 was not affected by mtiRL knockdown (Figure S6, Supporting Information). To determine whether mtiRL also binds to Yes1, we conduced RNA pull‐down assay in Cd‐SV‐HUC‐1 cells. The result showed no interaction between mtiRL and Yes1 (Figure S7, Supporting Information). We speculated that mtiRL combines with ANXA2 to change its spatial conformation and improve the interaction between ANXA2 and Yes1, leading to an increase in the level of p‐ANXA2‐Y24. Mao et al.^[^ ^28^ ^]^ recently reported that Yes1 activates ANXA2 at Tyr24 and that the overexpression of wild‐type, but not mutant ANXA2 (Tyr24F), restores the invasion and migration of Yes1‐KD GC cells, suggesting that p‐ANXA2‐Y24 promotes GC progression. In our study, we also revealed that the overexpression of ANXA2 could rescue the proliferative ability of mtiRL‐KD Cd‐SV‐HUC‐1 cells and that the level of p‐ANXA2‐Y24 was high, correlating with poor prognosis in BC, suggesting that p‐ANXA2‐Y24 facilitates BC development.

Emerging data have revealed that tRNA modifications affect tsRNA production. The m^5^C modification inhibits the binding of ANG to methylated tRNA, thereby increasing tRNA stability and reducing its production.^[^ ^36^, ^37^ ^]^ In addition to m^5^C, N1‐methyladenine (m^1^A), N3‐methylcytidine (m^3^C), and N5‐methyluracil (m^5^U) suppress ANG‐mediated tRNA cleavage and decrease tsRNA biogenesis.^[^ ^38^, ^39^, ^40^ ^]^ Another rich tRNA modification, ψ, mediated by PUS7, boosts 18 nt terminal oligoguanine tRF‐5 production and hampers translation initiation by displacing the eIF4F complex, further affecting hematopoietic functions.^[^ ^41^ ^]^ Some tRNAs containing m^7^G at position 46 stabilize the tertiary tRNA fold by enhancing the geometrical structure of the N13–N22–m^7^G46 base triplet.^[^ ^42^ ^]^ A recent study has demonstrated that the abundance of 5′tRFs originating from METTL1‐target tRNA Cys and Ala, rather than other METTL1‐tRNA targets is significantly elevated in METTL1 downregulated PC3, DU145, and 22Rv1 cells. This finding suggests that the cleavage of METTL1 tRNA targets occurs selectively in the presence of hypomethylation.^[^ ^43^ ^]^ Another research revealed that there were no substantial disparities observed in the stability of fully mature transfer RNA (tRNA) within PC3 cells that lacked METTL1. Moreover, the findings of this research indicate that the formation of distinct stress‐induced 5′ terminal RNA fragments (5′tRFs) is influenced by the specific methylation catalyzed by METTL1.^[^ ^44^ ^]^ Here, we demonstrated that neither a decrease in m^7^G mediated by METTL1 knockout nor an increasing by METTL1 overexpression altered the total abundance of tsRNAs (Figure S8, Supporting Information).

Studies focused on methylated tsRNAs are scarce—except for those on m^5^C‐ and m^2^G‐modified tsRNAs, m^1^A‐modified tRF‐3b, and Ψ‐modified tsRNAs.^[^ ^12^, ^13^, ^14^ ^]^ Furthermore, the biological function of m^7^G‐modified tsRNA remains unclear. In this study, we analyzed the trend in tsRNA alterations in multistage CdCl_2_‐treated urothelial cells and the level of m^7^G‐modified tsRNAs using an Arraystar Human m^7^G small RNA modification microarray after METTL1 knockout. Ultimately, we identified a new m^7^G‐modified tsRNA, mtiRL. We further extracted endogenous mtiRLs and evaluated their functions and regulatory mechanisms.

In summary, our study not only uncovered a novel mechanism of tsRNA epigenetic regulation in BC malignancy, but also provides a new strategy for tumor therapy.

## 4 Experimental Section

### Cell Culture and Construction of Multistage CdCl_2_‐Induced Transformed Malignant Cell Line

Human uroepithelial cells (SV‐HUC‐1) were purchased from the American Type Culture Collection (Manassas, VA, USA) and maintained in the F‐12K medium with 10% FBS (Gibco). The BCa cell line T24 was acquired from the Institute of Cell Biology, Chinese Academy of Sciences (Shanghai, China), and cultured in RPMI 1640 supplemented with 10% FBS. To construct CdCl_2_‐induced transformed malignant cell lines, SV‐HUC‐1 cells were separately treated with CdCl_2_ (Sigma–Aldrich, final concentration: 10 mg mL^−1^) for 2, 4, or 6 weeks. All cells were grown at 37 °C in a 5% CO_2_ humidified incubator.

### LC‐MS‐Based tRNA Modification

The tRNA modifications were analyzed using LC/MS. tRNA was extracted from the total RNA samples by the NEBNext Poly(A) tRNA Magnetic Isolation Module (NEB, E7490) and quantified by the Qubit RNA HS Assay kit (ThermoFisher, Q32855). tRNA was then hydrolyzed to single dephosphorylated nucleosides using an enzyme mix. LC‐MS analysis was conducted on an Agilent 6460 QQQ mass spectrometer with an Agilent 1260 HPLC system in multi‐reaction monitoring detection mode. The LC‐MS/MS testing was conducted by Kangcheng Bio‐tech (Shanghai, China).

### Small RNA Sequencing

Total RNA was extracted and pre‐treated to remove RNA modifications that interfered with the construction of the small RNA‐seq library. Then, 3′ and 5′ small RNA adapters were sequentially ligated to total RNA. cDNA was synthesized and amplified using Illumina proprietary RT primers and amplification primers. Subsequently, ≈ 134–160 bp PCR‐amplified fragments were obtained from the PAGE gel. The libraries were diluted, loaded onto a reagent cartridge, and subjected to a sequencing run on an Illumina NextSeq 500 system using the NextSeq 500/550 V2 kit (#FC‐404‐2005, Illumina), according to the manufacturer's instructions. Differentially expressed tRFs and tiRNAs were screened based on count values using the R package edgeR. Small RNA sequencing was performed and analyzed by Kangcheng Biotech (Shanghai, China).

### Arraystar Human m^7^G Small RNA Modification Microarray

Arraystar Human m^7^G small RNA modification microarray analysis was performed by Kangcheng Biotech (Shanghai, China). Briefly, 1–5 µg of each total RNA sample was immunoprecipitated using 6 µg of an anti‐m^7^G antibody (MBL, RN017M) with 1 mg of Protein G Dynabeads (Thermo Fisher, 11203D) in 500 µL of MeRIP buffer. The modified RNAs were extracted from the anti‐m^7^G antibody‐coated magnetic beads as the “IP” sample (Cy5‐labeled). The supernatant contained the unmodified RNAs as the “Sup” sample (Cy3‐labeled). The labeled RNAs were combined and hybridized onto an Arraystar Human Small RNA Microarray (8 × 15 K, Arraystar). After washing, the arrays were scanned based on two‐color channels using an Agilent Scanner G2505C. The array images were analyzed using Agilent Feature Extraction software (version 11.0.1.1). The average of the log_2_‐scaled spike‐in RNA intensities was used to normalize the IP and Sup data. Then, “m^7^G abundance” was analyzed based on the Cy5‐labeled IP (modified RNA)‐normalized intensities. Differentially expressed m^7^G‐methylated RNAs between the two groups were identified using the FC and statistical significance (p‐value) thresholds.

### m^7^G IP Coupled With Stem‐Loop Quantitative Real‐Time Reverse Transcription PCR

An m^7^G IP method coupled methos was developed with a stem‐loop RT‐qPCR method to specifically quantify m^7^G‐modified 3′‐tiRNA. First, total RNA was extracted by TRIzol reagent and incubated with an appropriate amount of an anti‐m^7^G antibody for 3 h. Then, protein A/G magnetic beads were added and incubated overnight at 4 °C. The m^7^G‐binding RNA was extracted from the beads, followed by treatment with T4 PNK and ATP to phosphorylate the 5′ end of the 3′ tiRNA. Subsequently, a 5′ RNA adaptor was ligated to the 3′ tiRNA. A specific stem‐loop reverse transcription primer was designed for the 3′‐tiRNA, followed by reverse transcription using the PrimeScript RT Reagent Kit (Takara, RR037A), according to the manufacturer's instructions. PCR primers were designed to amplify a region across the adaptor ligation sites. qPCR was performed using the ChamQ SYBR qPCR Master Mix (Vazyme). The details of stem‐loop RT primers and qPCR primers are provided in Table S1 (Supporting Information).

### Capture of Endogenous m^7^G‐Modified tiRNAs

As previously described,^[^ ^45^ ^]^ small RNA was extracted using a PureLink miRNA isolation kit (Invitrogen). tiRNAs were purified on 15% TBE‐urea polyacrylamide gels and soaked in elution buffer, followed by hybridization with biotinylated oligonucleotide DNA probes complementary to the target tiRNAs. Then the biotinylated oligo‐tiRNA complex was bound to Streptavidin Magnetic Beads (Thermo Scientific, 88 816) and extracted using trziol regent. The DNA oligo probe was digested using 10 U of DNase I (New England Biolabs). Captured endogenous tiRNAs were used for IP with an m^7^G antibody. Endogenous m^7^G‐modified tiRNAs were also identified. The sequences of the DNA oligoprobes are listed in Table S2 (Supporting Information).

### Northern Blotting

Northern blotting was performed as previously described.^[^ ^21^ ^]^ Small RNA was isolated and electrophoresed on 12% TBE‐urea polyacrylamide gels, followed by transfer onto nylon membranes. The membranes were then hybridized with digoxigenin (DIG)‐labeled DNA probes and incubated with an anti‐DIG antibody (Roche, #11 093 274 910). Signals were detected using a ChemiDoc imaging system. The probe sequences are listed in Table S2 (Supporting Information).

### RNA Pulldown

Endogenous m^7^G‐modified tiRNAs were extracted as described previously and labeled endogenous m^7^G‐tiRNAs using the Thermo Scientific Pierce RNA 3′ Desthiobiotinylation Kit. RNA pull‐down was conducted according to the manufacturer's instructions. The labeled RNA was bound to Streptavidin Magnetic Beads. RNA–protein mixtures were added to the RNA‐bound beads and incubated for 1 h at 4 °C. The RNA‐binding protein complexes were pulled down for mass spectrometry or western blot analysis.

### Silencing and Overexpression of 3′tiRNA Using an Inhibitor or Mimic

The three RNA inhibitors and mimics were synthesized by Sangon Biotech (Shanghai, China). Sequences of the inhibitors and mimics are listed in Table S2 (Supporting Information). For transfection, the Lipofectamine RNA iMAX reagent was used according to the manufacturer's instructions. RNA was collected after 48 h to detect the effects of the inhibitors or mimics.

### Animal Experiments

To construct multiorgan carcinogenesis model, the 5‐week‐old female ICR mice were randomly divided into two groups. Mice were intraperitoneally injected with Benzopyrene or sterile edible oil once a week for 6 weeks. After 5 months mice were sacrificed, and the tumors were dissected.

To establish multi‐stage bladder cancer models, the 6‐week‐old female SD rats were randomly grouped. After one week of adaptation, anesthesia in rats was induced with 3% isoflurane gas and 1.5% isoflurane for maintenance. Subsequently, rats were given weekly intravesical perfusion of CdCl_2_ (100 mg k^−1^g) or sterile PBS (pH 7.4) for 6 weeks. Rats were euthanized at 4 weeks, 8 weeks, and 12 weeks to collect bladder tissues for HE‐staining, immunohistochemical (IHC) staining and the level of mtiRL testing.

To induce tumor formation, 2 × 10^6^ T24 cells were subcutaneously injected into 5‐week‐old BALB/cJNju‐Foxn1nu/Nju nude mice (Nanjing Biomedical Research Institute, Nanjing University, China). One week later, the tumors were treated with an NC antagomir, tiRL antagomir, NS antagomir, and tiRL agomir via multisite intratumoral injection five times every 3 days. Tumor volumes were measured weekly, and the tumors were extracted after 4 weeks. The NC antagomir, tiRL antagomir, NS antagomir, and tiRL agomir were synthesized by Sangon (Shanghai, China). The sequences are listed in Table S2 (Supporting Information). All animal assays were performed in accordance with the institutional guidelines for animal care and use. Animal experiments were approved by the Institutional Ethics Committee for Clinical Research and Animal Trials of the First Affiliated Hospital of Sun Yat‐sen University ([2017]257).

### Rat Organoid Culture

Rat organoid culture assay was performed according to the reference.^[^ ^46^ ^]^ The organoid culture media produced by STEMCELL Technologies.

### In Vitro Kinase Assay

An in vitro kinase assay was conducted as described previously.^[^ ^28^ ^]^ Recombinant human His‐ANXA2 (100 ng; Jingxin Bio, #GXP87073) was incubated with recombinant human active GST‐Yes1 (100 ng; MCE, # HY‐P73486) and mtiRL (1 µg) at 30 °C for 30 min in reaction buffer (20 mM HEPES (pH 7.6), 20 mM MgCl_2_, 0.2 mM ATP, 2 mM DTT, 20 mM β‐glycerophosphate, 0.1 mM sodium orthovanadate). The reaction was stopped by adding 30 µL of SDS‐PAGE sample buffer followed by immunoblotting.

### Statistical Analysis

Statistical analyses were conducted using GraphPad Prism software (GraphPad Prism). All experiments were independently repeated at least three times. Data are presented as the mean ± standard error of the mean. Statistical comparisons were performed using the two‐sided unpaired Student's *t*‐test or repeated‐measures ANOVA. Statistical significance was set at *p* < 0.05.

### Availability of Data and Materials

The small RNA sequencing data generated in this study were deposited in the GSA under the accession number HRA006533. Arraystar Human m^7^G small RNA modification microarray data in this study were deposited in Gene Expression Omnibus (GEO) under accession number GSE232579.

## Conflict of Interest

The authors declare no conflict of interest.

## Author Contributions

X.Y. and W.H. contributed equally to this work. X.L.Y. designed and performed the experiments. X.L.Y., W.Y.H., and Y.P.H. performed the animal experiments. Y.L. and D.J. performed in vitro assays. C.C. and B.T.Y. were responsible for the RIP assays. C.C.Z. and Y.M.L. performed WB assays. H.Q.Z. and M.R.L. organized data. W.D.J., W.Q.W., and G.Y. conceived the project and critically revised the manuscript. All authors read and approved the final manuscript.

## Supporting information

Supporting Information

## Data Availability

The small RNA sequencing data generated in this study were deposited in the GSA under the accession number HRA006533. Arraystar Human m7G small RNA modification microarray data in this study were deposited in Gene Expression Omnibus (GEO) under accession number GSE232579.
