# Supplementary material for: A Novel tsRNA, m7G‐3′ tiRNA LysTTT, Promotes Bladder Cancer Malignancy Via Regulating ANXA2 Phosphorylation
Source: Adv Sci (Weinh). 2024 Jun 18;11(31):2400115. doi: 10.1002/advs.202400115 (PMC11336930; doi:10.1002/advs.202400115)
Supplement: Supplementary file 1 — Supporting Information [file ADVS-11-2400115-s001.docx]

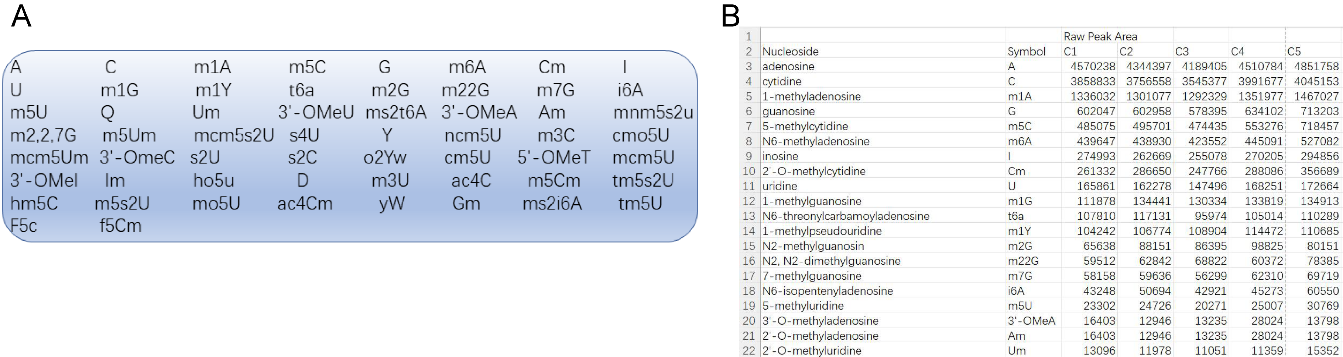


**Figure S1.** **tRNA modification was analyzed using LC/MS in multistage CdCl_2_ malignant transformed cells.** (A) Modification profiles of tRNA from multistage CdCl_2_ malignant transformed cells. (B) tRNA modifications with higher relative abundance. C1: Human uroepithelial cells (SV-HUC-1); C2: Human uroepithelial cells (SV-HUC-1) treated with CdCl_2_ for 2 weeks; C3: Human uroepithelial cells (SV-HUC-1) treated with CdCl_2_ for 4 weeks; C4: Human uroepithelial cells (SV-HUC-1) treated with CdCl_2_ for 6 weeks; C5: CdCl2 malignant transformed cells (Cd-SV-HUC-1).

**
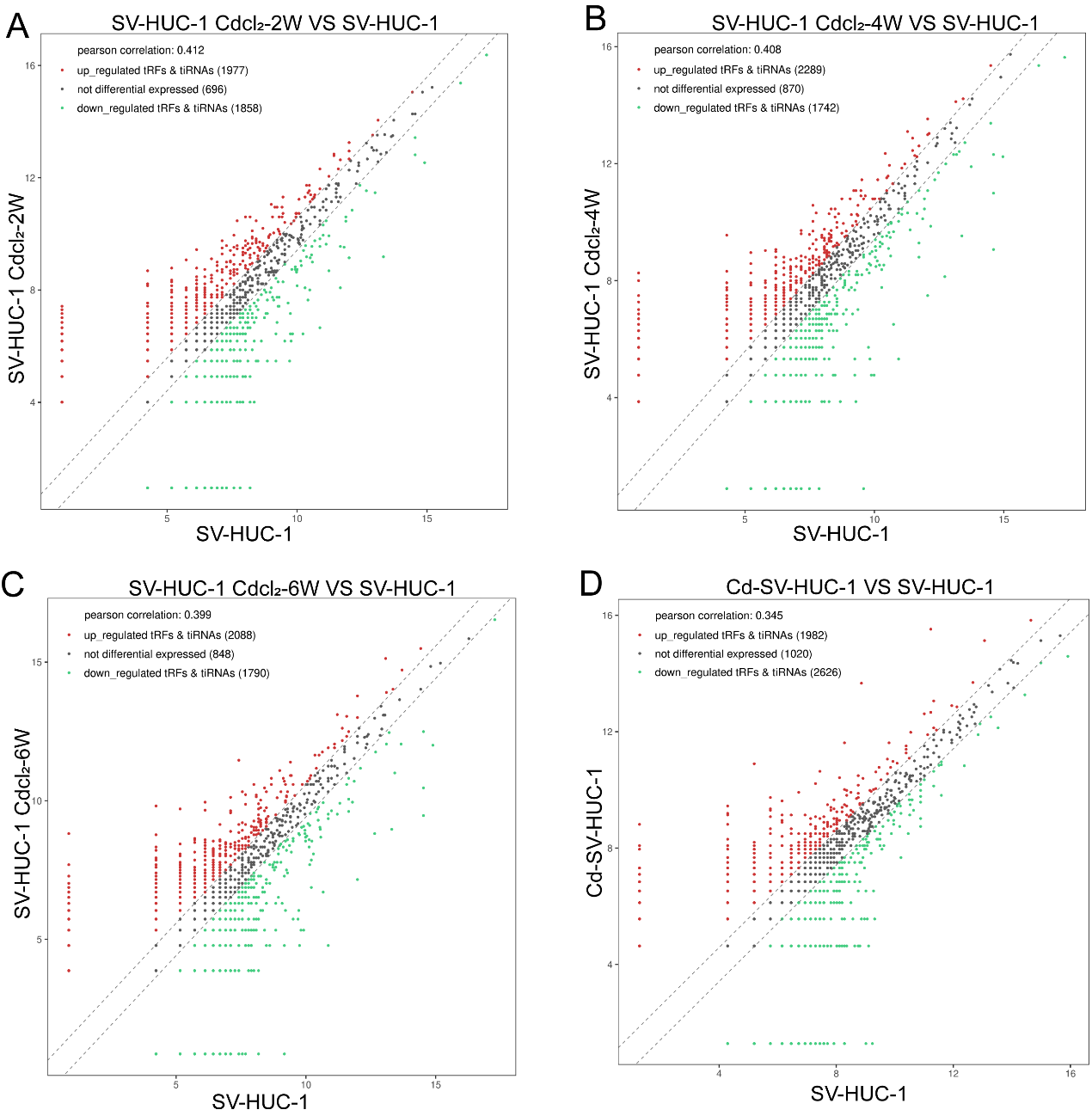
**

**Figure S2.** **Differentially expressed tRFs and tiRNAs (fold change ≥ 2 or < 0.5 and p-value < 0.05) in multistage CdCl_2_ malignant transformed cells compared to human uroepithelial cells (SV-HUC-1).**

**
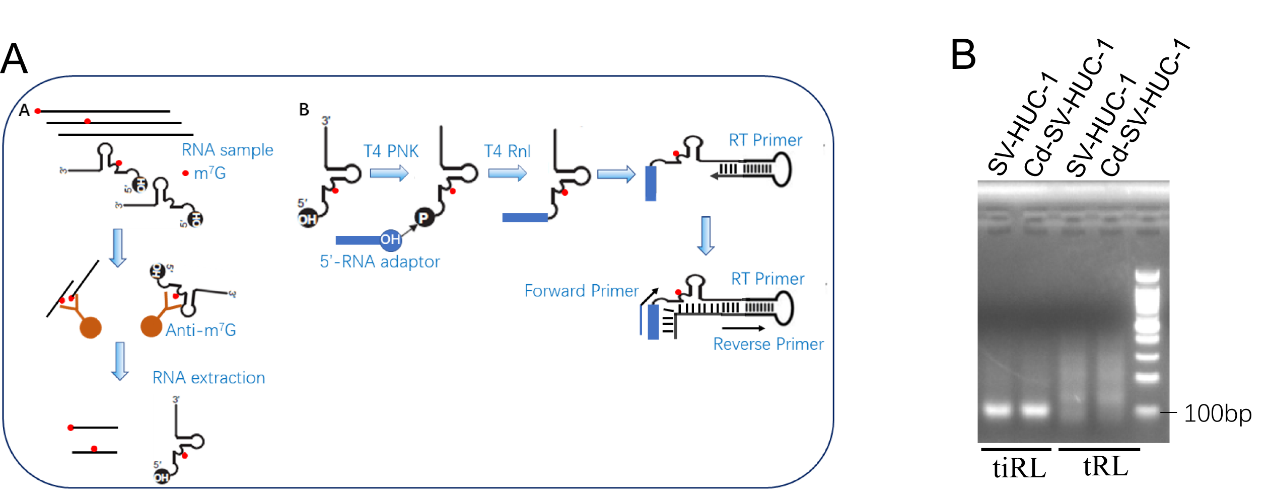
**

**Figure S3.** **Stem-loop RT PCR method that could quantify specifically expression of 3′tiRNA tRNA.** (A) Schematic illustration of Stem-loop RT PCR method. (B) Agarose electrophoresis of tiRL and tRL products. tiRL: 3′tiRNA Lys^TTT^; tRL: tRNA Lys^TTT^.

**
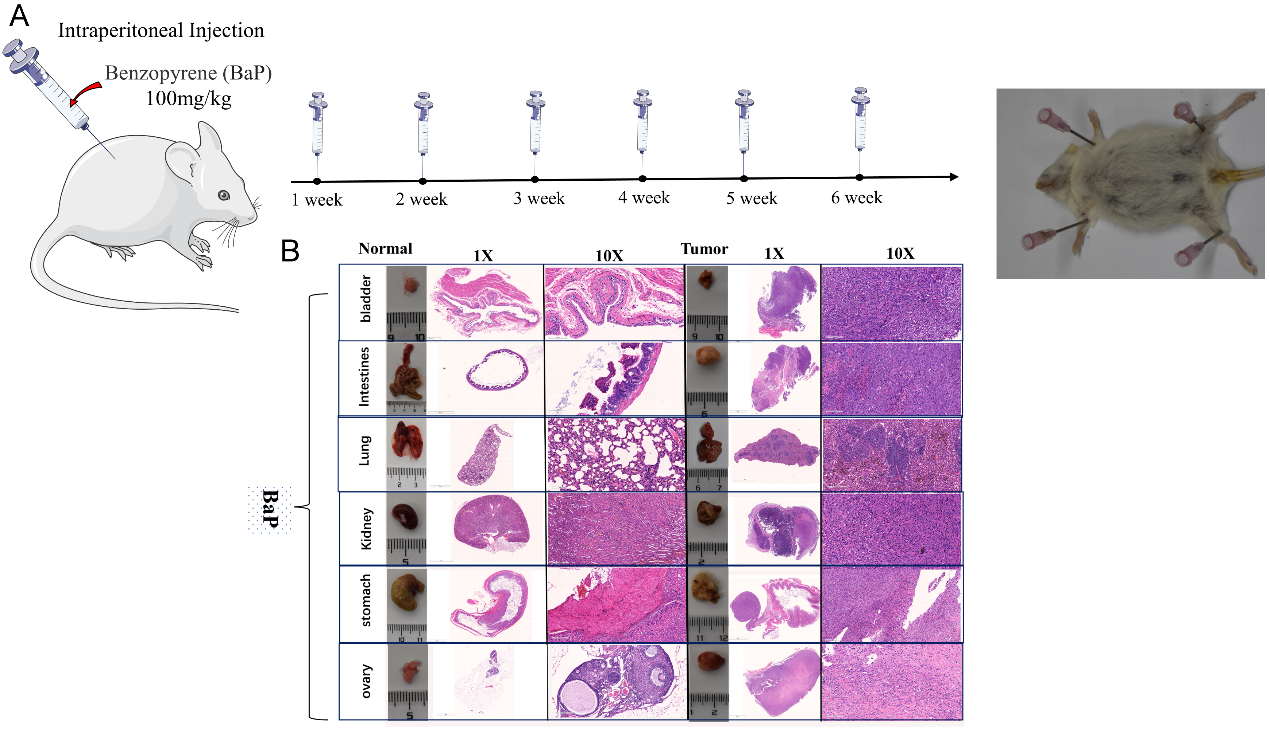
**

**Figure S4.** **mouse models of benzopyrene-induced multiple-organ carcinogenesis.** (A) Schematic illustration of benzopyrene-induced multiple-organ carcinogenesis. (B) multiple-organ tumors were analyzed by HE staining.


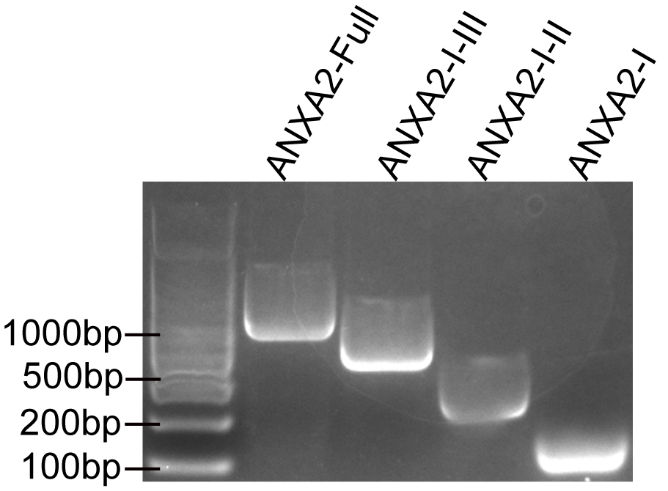


**Figure S5.** **Construction of vectors carrying GFP-tagged full-length (domain I-IV (1-339aa)) and truncated ANXA2**.


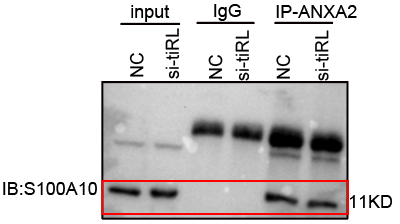


**Figure S6.** **Co-immunoprecipitation (co-IP) showed that the interaction between ANXA2 and S100A10 was not affect after mtiRL knockdown in Cd-SV-HUC-1 cells.**

**
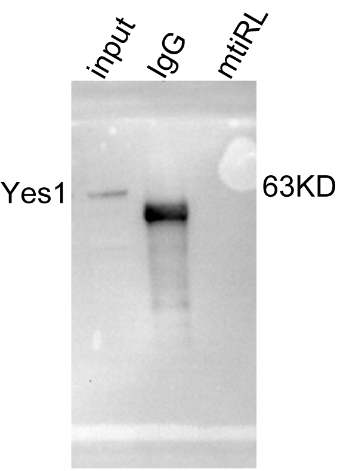
**

**Figure S7.** **RNA pull-down showed that** **no interaction between mtiRL and Yes1 were found in Cd-SV-HUC-1 cells.**

**
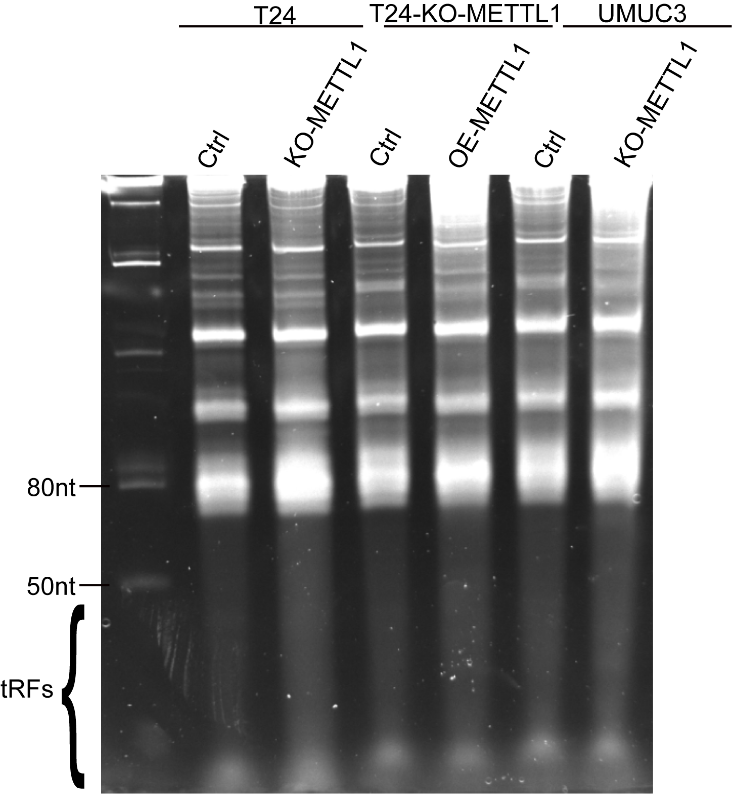
**

**Figure S8. Neither METTL1 knockout nor METTL1 overexpression changed the total abundance of tsRNAs.**

**Table S1.** The sequences of stem-loop RT primer and qPCR primer

| **Name** | **sequence** |
| --- | --- |
| Human-3'tiRNA-Lys^TTT^-Rev-Prime | CTCAACTGGTGTCGTGGAGTCGGCAATTCAGTTGAGCGCCCAAA |
| Mice/Rat-3'tiRNA-Lys^TTT^ -Rev-Prime | CTCAACTGGTGTCGTGGAGTCGGCAATTCAGTTGAGCGCCCGAA |
| Human-3'tiRNA-Lys^TTT^-F | ACACTCCAGCTGGGACAGTCCGACGATCTTTAACCTGAG |
| Mice/Rat-3'tiRNA-Lys^TTT^ -F | ACACTCCAGCTGGGACAGTCCGACGATCTTAATCTGAGG |
| U6 RT | TTCACGAATTTGCGTGTCAT |
| U6 forward | CGCTTCGGCAGCACATATAC |
| Universe reverse primer | CTCAACTGGTGTCGTGGAGTCGGC |
| 5′-RNA adaptor | ACAGUCCGACGAUC |
| ANXA2 -gRNA-1 | TCGTAGGATCTCTATGACGC |
| ANXA2 -gRNA-2 | ATTATATCCAGGTAAGCCCG |
| ANXA2 -gRNA-3 | GTCCCTGTACTATTATATCC |
| Rat-ANXA2-gRNA | ACACCAACTTCGACGCTGAG |

**Table S2.** The sequences of probes used in this study

| **Name** | **sequence** |
| --- | --- |
| Inhibitor-3'tiRNA-Lys^TTT^ | ACCCUGAACCCUCAGGUUAA |
| mimic-3'tiRNA-Lys^TTT^ | UUAACCUGAGGGUUCAGGGUUCAAGUCUCUGUUUGGGCGCCA |
| Biotin-3'tiRNA-Lys^TTT^ | ACCCTGAACCCTCAGGTTAA (5'- Biotin) |
| Dig-3'tiRNA-Lys^TTT^ | ACCCTGAACCCTCAGGTTAA (3'- digoxigenin) |
| Dig-U6 | TGGAACGCTTCACGAATTTG (3'- digoxigenin) |
| agomir -3'tiRNA-Lys^TTT^ | UUAACCUGAGGGUUCAGGGUUCAAGUCUCUGUUUGGGCGCCA  (2'- OMe) |
| NS agomir | CAGUACUUUUGUGUACAA |
| antagomir -3'tiRNA-LysTTT | UGGCGCCCAAACAGAGACUUGAACCCUGAACCCUCAGGUUAA  (2'- OMe) |
| NC antagomir | UUGUACUACACAAAAGUACUG |

**Table S3.** The sequences of 3'tiRNA-Lys^TTT^

| **Name** | **sequence** |
| --- | --- |
| Human-3'tiRNA-Lys^TTT^ | TTAACCTGAGGGTTCAGGGTTCAAGTCTCTGTTTGGGCGCCA |
| Mice-3'tiRNA-Lys^TTT^ | TTAATCTGAGGGtCCAGGGTTCAAGTCCCTGTTCGGGCGCCA |
| Rat-3'tiRNA-Lys^TTT^ | TTAATCTGAGGGtCCAGGGTTCAAGTCCCTGTTCGGGCGCCA |

**Supplementary Materials and Methods**

**Co-immunoprecipitation and Western blot**

Cells were lysed in ice-cold lysis buffer (50 mM Tris-HCl, pH 7.5, 250 mM/300 mM NaCl, 3 mM EGTA, 3 mM EDTA, 1% Triton X-100, 0.5% NP40, 10% glycerol, 2 mM DTT, 1 mM PMSF, 0.1 mM sodium vanadate, 2 mM PNPP, 1X Proteases inhibitors cocktail (5892953001, Roche) for 30 min, sonicated every 5 minutes and centrifuged at 12,000 × rpm for 10 min at 4 °C. The supernatant was collected, and protein concentration was detected by BCA protein assay kit (Takara).

For endogenous co-IP, cell lysates containing 1 mg of protein were incubated with specific antibodies (Anti-Yes1 antibody (Proteintech, #20243-1-AP); Anti-ANXA2 antibody (Proteintech, #11256-1-AP); IgG(Proteintech, #30000-0-AP) overnight at 4 °C, 50 μl of protein A/G magnetic beads (Thermo Scientific, 88803) were added into the lysates and incubated for 3h. Beads were then washed four times in lysis buffer, and then subjected to western blotting. For exogenous co-IP, HEK293T cells were co-transfected with FLAG -tagged ANXA2 and GFP-tagged Yes1. Anti -FLAG nanobody magarose beads were purchased from Kangti Life Science Technology Co. Ltd (Shenzhen，KTSM1334). Anti- FLAG antibody (Proteintech, #66008-4-Ig) and anti- GFP antibody (Santa Cruz Biotechnology, #sc-9996) were used. The next steps are the same as for endogenous co-IP protocol.

Cytoplasmic and nuclear protein was extracted using Cytoplasmic and Nuclear Protein Extraction Kits (Thermo Scientific, Waltham, MA) according to manufacturer’s instructions. For western blotting, the same amount samples were run on SDS-PAGE gels and transferred onto Immobilon-P PVDF membranes (Merck Millipore). After blocking with 5% BSA in TBST for 1 h, the membranes were incubated with primary antibodies (Anti-Yes1 antibody (Proteintech, #20243-1-AP); Anti-ANXA2 antibody (Cell Signaling Technology, #18235s); Phospho-Annexin A2 (Tyr24) antibody (1:200; Invitrogen, # PA5-105371); Anti- FLAG antibody (Proteintech, #66008-4-Ig) overnight at 4 °C and then the secondary antibody for 1h. Immunoreactive bands were visualized by the Enhanced Chemiluminescence (ECL) reagents (Thermo Fisher Scientific).

**Immunofluorescence**

Cells were fixed with 4% paraformaldehyde, blocked with 1% BSA, permeabilizated with 0.1% Triton X-100 for 20 min and processed for immunofluorescence. After blocking with 1% BSA in TBST for 1 h, and incubated overnight at 4 °C with anti-ANXA2 antibody (1:200; Proteintech, #11256-1-AP), anti-Yes1 antibody (1:200; Santa Cruz, # sc-48396) or Phospho-Annexin A2 (Tyr24) antibody (1:200; Invitrogen, # PA5-105371), followed by incubation with Alexa Fluor™ 488 goat anti-Rabbit IgG (H+L) Cross-Adsorbed Secondary Antibody(1:1000; A-11008; Invitrogen), or Alexa Fluor 568 donkey anti-rabbit IgG (H+L) (1:1000; A-11011; Invitrogen). Nuclear staining was performed using 1× 4′,6-diamidino-2-phenylindole (S2110; Solarbio, Beijing, China), and images were obtained with a confocal microscope (LSM880; Carl Zeiss, Oberkochen, Germany).

**Immunohistochemistry (IHC) assay**

For IHC, human tissues were incubated at 65 °C for 30 min and then rehydrated in a gradient of ethanol. Endogenous peroxidase was blocked using 3% H_2_O_2_, followed by antigen retrieval. Thereafter, the tissues were incubated with primary antibodies (Phospho-Annexin A2 (Tyr24) antibody (1:200; Invitrogen, # PA5-105371; Anti- cytokeratin 5 (1:200; Abcam, #ab5263S; Ki67 (1;200; Cell Signaling Technology, # 12202S)) at 4 °C overnight after blocking with 5% BSA buffer for 30 min. A semiquantitative scoring system was used to assess IHC staining, as previously described [1]

**Proliferation and** **migration assays**

Proliferation and migration assays were performed as previously described [1]. Cell lines were seeded in 96-well plates at concentration of 2000 cells/well and incubated for 0–5 days. Cell proliferation was tested using a CellTiter 96 AQueous One Solution Cell Proliferation Assay Kit (Promega).

For the migration assay, cells were grown in 96-well Essen ImageLock plates (Essen Bioscience) at a density of 1 × 10^5^/well. The scratch assays were performed using a real-time cell imaging system. Data were evaluated based on the relative wound density and analyzed using GraphPad Prism software.

References

[1] Jin H, Ying X, Que B, Wang X, Chao Y, Zhang H, Yuan Z, Qi D, Lin S, Min W,et al. N(6)-methyladenosine modification of ITGA6 mRNA promotes the development and progression of bladder cancer. EBioMedicine. 2019; 47:195-207.
